# Post-infectious Moyamoya Syndrome: A Review of Existing Scientific Literature From 2000 to 2023

**DOI:** 10.7759/cureus.63643

**Published:** 2024-07-02

**Authors:** Haseeb Mehmood Qadri, Raahim A Bashir, Arham Amir, Maira Jabbar Chaudhry, Muhammad Farhad Alam, Usama Afraz Younas, Asif Bashir

**Affiliations:** 1 Neurological Surgery, Punjab Institute of Neurosciences, Lahore, PAK; 2 General Surgery and Surgical Oncology, Shaikh Zayed Medical Complex, Lahore, PAK; 3 Pathology, Azra Naheed Medical College, Lahore, PAK; 4 Neurology, Shaikh Zayed Medical Complex, Lahore, PAK; 5 Surgery, Jinnah Hospital, Lahore, PAK

**Keywords:** post-infectious, infectious, moya moya syndrome, syndrome, moyamoya

## Abstract

Moyamoya disease (MMD) is considered a primary disorder of an unknown etiology. In contrast, Moyamoya syndrome (MMS) refers to MMD associated with other underlying diseases, such as meningitis in childhood, neurofibromatosis type II, Down syndrome, cranial irradiation, and different types of anemias, particularly hemoglobinopathies. We aimed to provide a comprehensive clinicopathological overview of MMS. All case reports and case series published from 2000 to 2023 pertaining to MMD were included in the study. Case studies, original articles, editorials, letters to editors, and clinical images were excluded. The search was conducted using the Boolean operators ("AND" and "OR") on PubMed and Google Scholar. A total of 13 case reports and one case series study were included.

The study suggests infection might be a trigger in susceptible individuals. The autoimmune antibody findings (anti-double stranded DNA IgG) suggest a potential autoimmune component in some cases. There were diverse presentations and outcomes of post-infectious MMS, with a striking predominance of pediatric cases (66.66%) and a possible female predominance. Both computerized tomography (CT) and magnetic resonance imaging (MRI) showed evidence of restricted blood flow. CT showed that stenosis, occlusion, and collateral formation were frequent vascular findings, but often unspecified in severity. Infarction, hypodensities, and hematoma were the most common parenchymal findings (22.22% each). The findings on MRI were stenosis (50%) and collateral formation (44.44%). Infarction was the most common finding (66.66%) in parenchyma. Hydrocephalus, encephalomalacia, and atrophy were less frequent. Lesions were most frequent in the internal carotid artery (66.66%), middle cerebral artery (66.66%), and anterior cerebral artery (50%). Lesions were less frequent in the posterior cerebral, vertebral, and basilar arteries. The frontal lobe (38.89%) and basal ganglia (33.33%) were commonly affected parenchymal regions. The most common risk factor was human immunodeficiency virus (HIV) infection (50%), followed by trisomy 21, cryptococcal, and other types of meningitides. Aspirin (50%) and antiretroviral therapy (38.89%) were the cornerstones of treatment for MMS.

This review accentuates the noteworthy obstacles presented by post-infectious MMS, especially its catastrophic effect on children and its correlation with HIV/AIDS. According to our elaborate literature search using PubMed and Google Scholar, this is the first narrative review in the existing scientific literature summarizing the literature on post-infectious MMS.

## Introduction and background

Moyamoya disease (MMD) is a cerebrovascular steno-occlusive condition characterized by endothelial hyperplasia and fibrosis of the intracranial portion of the carotid artery and its proximal branches, leading to progressive stenosis and occlusion of the terminal portion of the internal carotid artery. This results in the formation of an abnormal network of dilated, fragile perforators at the base of the brain, often clinically manifesting as ischemic or hemorrhagic stroke with high rates of morbidity and mortality [1]. In 1969, Suzuki et al. introduced the term "moyamoya" (a Japanese word for "puff of smoke" or "hazy") to describe the tangled appearance of tiny vessels compensating for the blockage according to the cerebral angiographic findings [2].

MMD is a relatively rare condition but has documented cases worldwide. It is most prevalent in East Asian countries, particularly Japan and Korea, and among individuals of Chinese descent, where the annual incidence is estimated to be around 0.35 to 0.94 per 100,000 individuals [1,3]. However, cases have been reported in various other parts of the world, including North America, Europe, and many Asian countries. This disease most commonly presents in children and young adults and has a bimodal age distribution, with peaks around 5-10 and 30-40 years old [1]. In two large studies of MMD from Japan, the women-to-men ratio was up to 1.9:1, and among these, 11%-12% had a family history of MMD, suggesting a genetic predisposition [3].

In the United States, a study from the National Inpatient Sample between 2002 and 2008 showed the race distribution was 49% White, 24% Black, and 11% Asian, with the ethnic distribution being Hispanic in 11%. In another study from the National Inpatient Sample between 2005 and 2008, MMD diagnosis was more frequent in White women [3]. The incidence of MMD may be increasing due to improved diagnostic techniques and accuracy, noninvasive radiological techniques, increased awareness among healthcare professionals, and better access to healthcare services [1,4].

MMD is considered a primary disorder of unknown etiology. However, Moyamoya syndrome (MMS) refers to MMD secondary to an underlying disease, such as meningitis, neurofibromatosis type II, Down syndrome, cranial irradiation, and different types of hemoglobinopathies [2]. It is also known as "quasi-moyamoya" in Eastern countries [3]. These underlying conditions or causes for MMS can include infections (cytomegalovirus infections, cerebral tuberculosis, meningitis), genetic conditions (sickle cell disease, thalassemia, Down syndrome, neurofibromatosis type 1, Marfan syndrome), radiation therapy to the head, brain tumors (meningioma, hemangioblastoma, craniopharyngioma, glioma), autoimmune diseases (systemic lupus erythematosus, antiphospholipid syndrome, polyarteritis nodosa, Sjogren syndrome), heart, renal, neurological disorders, and acquired conditions [3,5].

The goal of our narrative review is to bring awareness about infections as an emerging cause of MMS. Historically, MMS has been attributed to genetic and vascular factors, and therefore research has focused on these areas. However, as medical understanding progresses and more cases emerge where infections may be implicated, we are prompted to explore the association more rigorously. The identification of infection as an etiological factor highlights the complex interplay between infectious agents, immune responses, and vascular health, prompting further research into preventive strategies and therapeutic interventions targeting both infection and vascular inflammation. According to our extensive literature search using PubMed and Google Scholar, this is the first narrative review in the existing scientific literature summarizing the literature on post-infectious MMS.

## Review

Methodology

Background

This narrative review is structured to provide a comprehensive overview of MMS and MMD. We focused on different clinical presentations by synthesizing relevant data aimed at addressing trends in both diagnostic and treatment modalities.

Search Strategy

A thorough search for this narrative review was conducted using the database engines PubMed Central and Google Scholar. Keywords related to post-infective MMD and its clinical aspects were utilized to identify pertinent studies. The search was conducted using the Boolean operators ("AND" and "OR") with the following combination of keywords: “Moyamoya Syndrome”, “infection”, “Post infectious Moyamoya syndrome”, “Post infection Moyamoya Syndrome”, “Post infection Moyamoya”, “Moyamoya Syndrome after infection”, “Post Cryptococcal Moyamoya ” “HIV and Moyamoya Syndrome”, “Hepatitis and Moyamoya Syndrome”, “Cytomegalovirus and Moyamoya Syndrome”, “Moyamoya Syndrome and Tuberculosis meningitis”, “Moyamoya Syndrome and meningitis”, “Moyamoya Syndrome and Pyogenic meningitis”, Post-pneumococcal Moyamoya Syndrome”, “Varicella virus and Moyamoya Syndrome”, and “Herpes Zoster and Moyamoya Syndrome” were used.

Inclusion Criteria

All case reports and case series published from 2000 to 2023 were included.

Exclusion Criteria

Case studies, original articles, letters to editors, editorials, and clinical images were excluded.

Data Extraction and Synthesis

Eighteen studies were selected to work on the set objective. Key information from the selected studies, including study design, sample size, intervention methods, outcomes, and conclusions, was extracted by seven reviewers who read all articles independently. The findings were then synthesized to identify common themes, trends, and gaps in knowledge. Descriptive analysis was employed to compile the results.

Results

All 18 studies were conducted between 2006 and 2023. All except one study were case series; the sole case series included a total of five new cases and two previously published cases. The studies spanned the globe with variability in age and gender. The most common objective in these studies was to identify the post-infective changes that lead to the disease, along with changes that could be observed by radiological methods and post-treatment. About 72.22% of the study articles were case reports, and 27.78% were case series (Table 1).

**Table 1:** The study design and number of studies conducted with percentage.

Study Articles	Number of Cases, n (N= 18)	Percentage Occurrence, %
Case reports	13	72.22%
Case series	5	27.78%

A clear predominance of pediatric cases in the reported instances of post-infectious MMS was seen and females (55.56%) were slightly more affected than males (Tables 2, 3).

**Table 2:** Age distribution and percentage occurrence, where the total number of patients N=18.

Age Group	Number of Cases, n	Percentage Occurrence, n%
Adults	6	33.33%
Pediatric	12	66.66%

**Table 3:** Gender distribution with percentage occurrence, where the total number of patients N=18.

Gender	Number of Cases, n	Percentage Occurrence, n%
Male	8	44.44%
Female	10	55.56%

A bimodal distribution of disease exists with occurrence in adults as well as pediatric populations (Table 4). 

**Table 4:** Mean age and standard deviation of adult and pediatric patients.

Age Group	Mean Age, Years	Standard Deviation
Adults	50.33	10.19
Pediatric	6.01	3.48

The most common symptoms at presentation were hemiplegia/hemiparesis (44.44%), fever (33.33%), headache (27.78%), and altered sensorium (27.78%) (Table 5).

**Table 5:** Clinical manifestations at presentation reported with frequency and percentage occurrence, where the total number of patients N=18.

Symptoms at Presentation	Number of Cases, n	Percentage Occurrence, n%
Hemiplegia/hemiparesis	8	44.44%
Fever	6	33.33%
Headache	5	27.78%
Altered sensorium	5	27.78%
Tingling/numbness	2	11.11%
Fits	2	11.11%
Neck stiffness	2	11.11%
Transient ischemic attack	1	5.56%
Behavioral issues	1	5.56%
Ear pain	1	5.56%
Generalized weakness	1	5.56%
Involuntary/irregular muscle movement	1	5.56%
Back pain	1	5.56%
Sore throat	1	5.56%
Speech disorder	1	5.56%
Shortness of breath	1	5.56%

The most frequent specified signs at presentation were hemiplegia and aphasia (each 11.11%). The most prevalent risk factor was HIV/AIDS (50% of cases). Several other risk factors included chromosomal abnormalities/trisomy 21, cryptococcal meningitis, and other types of meningitis (each 11.11%). A portion of cases had no identified risk factors (11.11%) (Tables 6, 7).

**Table 6:** Findings at physical examination of reported patients, where the total number of patients N=18.

Findings at Physical Examination	Number of Cases, n	Percentage Occurrence, n%
Unspecified	12	66.67%
Hemiplegia	2	11.11%
Aphasia	2	11.11%
Babinski positive bilaterally	1	5.56%
Hyposthenia	1	5.56%
Spasticity	1	5.56%
Hemianopia	1	5.56%
Hemineglect	1	5.56%
Paroxysmal tachycardia	1	5.56%

**Table 7:** Risks found in reported patients, where the total number of patients N=18. HTN: hypertension.

Risk Factors	Number of Cases, n	Percentage Occurrence, n/N, %
HIV/AIDS	9	50.00%
Chromosomal/trisomy 21	2	11.11%
No risk factors	2	11.11%
Cryptococcal meningitis	2	11.11%
Immunosuppressive therapy	1	5.56%
Tuberculous meningitis	1	5.56%
Cerebral malacia	1	5.56%
Trauma/fall	1	5.56%
Pneumococcal meningitis	1	5.56%
HTN	1	5.56%
Varicella	1	5.56%
Non-specific meningitis	1	5.56%

In a significant portion of cases (44.44%), the time elapsed since presentation was unspecified. Among specified cases, presentation within days of symptom onset was the most common (33.33%). Presentations occurring weeks, months, or years after symptom onset were less frequent (Table 8).

**Table 8:** Duration of symptoms before presentation, where the total number of patients N=18.

Time Elapsed Since Presentation	Number of Cases, n	Percentage Occurrence, n%
Unspecified	8	44.44%
Days	6	33.33%
Months	2	11.11%
Weeks	1	5.56%
Years	1	5.56%

Lesions were most frequent in the internal carotid artery (66.66%), middle cerebral artery (66.66%), and anterior cerebral artery (50%). Lesions were less frequent in the posterior cerebral, vertebral, and basilar arteries. The frontal lobe (38.89%) and basal ganglia (33.33%) were commonly affected parenchymal regions. Other brain regions, such as the temporal lobe, ventricles, occipital lobe, and parietal lobe, were affected less frequently (Table 9).

**Table 9:** Vascular and parenchymal sites involved with frequency and percentage occurrence, where the total number of patients N=18.

Site of Lesion	Number of Cases, n	Percentage Occurrence, n%
Vascular involvement		
Internal carotid artery	12	66.66%
Middle cerebral artery	12	66.66%
Anterior cerebral artery	9	50.00%
Posterior cerebral artery	3	16.67%
Vertebral artery	1	5.56%
Basilar artery	1	5.56%
Parenchymal involvement		
Frontal	7	38.89%
Basal ganglia	6	33.33%
Temporal	4	22.22%
Ventricles	4	22.22%
Occipital lobe	3	16.67%
Parietal	3	16.67%
Caudal nucleus	2	11.11%
Brain stem	2	11.11%
Ganglio-capsular	1	5.56%
Cellebellar	1	5.56%
White matter	1	5.56%
Thalamus	1	5.56%
Cerebral hemispheres	1	5.56%
Internal capsule	1	5.56%
Subcortex	1	5.56%

The vast majority of cases demonstrated bilateral involvement (77.78%). A smaller portion of cases exhibited right-sided lesions (22.22%). No cases were reported with solely left-sided lesions (Table 10).

**Table 10:** Laterality of lesions, where the total number of patients N=18.

Laterality	Number of Cases, n	Percentage Occurrence, n%
Bilateral	14	77.78%
Right	4	22.22%
Left	0	0.00%

Half of the cases had normal hematological findings (50%). A significant portion had unspecified hematological findings (33.33%). Less common findings included raised white blood cell count (particularly neutrophils), low CD4 count, and raised ASO titer/anti-DNase B (each at 5.56%) (Table 11).

**Table 11:** Hematological findings in reported patients, where the total number of patients N=18. ASO: anti-streptolysin O.

Hematological Findings	Number of Cases, n	Percentage Occurrence, n%
Normal	9	50.00%
Not specified	6	33.33%
Raised WBC, neutrophils	1	5.56%
Low CD4 count	1	5.56%
Raised ASO titer, anti-DNase B	1	5.56%

The majority of patients received medical management (77.78%). A smaller proportion of patients underwent either surgical intervention alone (11.11%) or a combination of medical and surgical treatment (11.11%) (Table 12).

**Table 12:** Type of management in reported patients, where the total number of patients N=18.

Type of Management	Number of Cases, n	Percentage Occurrence, n%
Medical	14	77.78%
Surgical	2	11.11%
Combined	2	11.11%

Both CT and MRI showed evidence of restricted blood flow. CT findings indicated that stenosis, occlusion, and collateral formation were frequent vascular findings, although often unspecified in severity. Infarction, hypodensities, and hematoma were the most common parenchymal findings (22.22% each). MRI findings showed that stenosis (50%) and collateral formation (44.44%) were the most prevalent vascular findings. Infarction was the most common parenchymal finding (66.66%). Hydrocephalus, encephalomalacia, and atrophy were less frequent (Table 13).

**Table 13:** Vascular and parenchymal findings on computerized tomography and magnetic resonance imaging, where the total number of patients N=18.

Radiology Findings	Number of Cases, n	Percentage Occurrence, n%
(1) Computerized tomography findings		
Vascular		
Unspecified diameter of occlusion	3	16.67%
Attenuation	1	5.56%
Near-occlusion	1	5.56%
Complete occlusion	1	5.56%
Saccular aneurysm	1	5.56%
Hypotrophy	1	5.56%
Parenchymal		
Infarct	4	22.22%
Hypodensities	4	22.22%
Hematoma	4	22.22%
Chronic lacunar infarct	1	5.56%
Calcification	1	5.56%
Encephalopathy	1	5.56%
Laminar necrosis	1	5.56%
(2) Magnetic resonance imaging findings		
Vascular findings		
Stenosis	9	50.00%
Collateral formation	8	44.44%
Attenuation	5	27.78%
Complete occlusion	4	22.22%
Enhancement	2	11.11%
Focal dilation	2	11.11%
Puff of smoke/abnormal vascular pattern	2	11.11%
Absence of signal void	1	5.56%
Poor filling	1	5.56%
Neovascularization	1	5.56%
Parenchymal		
Infarction	12	66.66%
Hydrocephalus	3	16.67%
Encephalomalacia	2	11.11%
Atrophy	2	11.11%
Hypodensities	2	11.11%
Unspecified lesions	2	11.11%
Hemorrhage	1	5.56%
Cystic lesions	1	5.56%
Hyperintensities	1	5.56%
Ivy sign	1	5.56%
Enhancement	1	5.56%
Dilated Virchow robin spaces	1	5.56%
Cerebral malacia	1	5.56%
Gliosis	1	5.56%

A significant portion of cultures lacked specific identification (38.89%). Normal culture findings were the next most common (16.67%). Several infectious agents were detected, including cryptococcal antigen (11.11%), unspecified Gram-positive cocci (11.11%), *Streptococcus pneumoniae* (5.56%), and VZV DNA + Serum-VZV IgM (5.56%). Additionally, antibodies for toxoplasma IgG and anti-double-stranded DNA IgG were present (5.56%) (Table 14).

**Table 14:** Reported findings on culture and sensitivity, where the total number of patients N=18.

Findings on Culture and Sensitivity Examination	Number of Cases, n	Percentage Occurrence, n%
Unspecified	7	38.89%
Normal finding	3	16.67%
CSF-crypotococcal antigen +	2	11.11%
CSF-gram-positive cocci (unspecified)	2	11.11%
CSF- *Streptococcus* *pneumoniae*	1	5.56%
CSF- VZV DNA + Serum-VZV IgM +	1	5.56%
Serum- toxoplasma IgG +	1	5.56%
Anti-double-stranded DNA IgG antibody +	1	5.56%

Aspirin (50%) and antiretroviral therapy (38.89%) were the cornerstones of treatment for MMS. The use of antiretroviral therapy highlights the significance of HIV/AIDS as a risk factor. Ceftriaxone (16.67%) was another commonly administered antibiotic, and a substantial portion (16.67%) had unspecified medications. Various antiplatelets, antibiotics, antifungals, and steroids were also used less frequently (Table 15).

**Table 15:** Medications used in medical management of reported patients, where the total number of patients N=18.

Medication Used	Number of Cases, n	Percentage Occurrence, n%
Aspirin	9	50.00%
Antiretroviral therapy	7	38.89%
Ceftriaxone	3	16.67%
Unspecified	3	16.67%
Antiplatelet	2	11.11%
Vancomycin	2	11.11%
Methylprednisolone	2	11.11%
Prednisolone	2	11.11%
Acyclovir	2	11.11%
Heparin	2	11.11%
Amphotericin B	1	5.56%
Fluconazole	1	5.56%
Dexamethasone	1	5.56%
Meropenem	1	5.56%
Rifampicin	1	5.56%
Valacyclovir	1	5.56%
Penicillin	1	5.56%
Ciprofloxacin	1	5.56%
Co-trimoxazole	1	5.56%

Treatment approaches were individualized. Medical management was most prevalent, often including aspirin and antibiotics. Antiretrovirals were essential for patients with HIV/AIDS. Surgery was less frequent, with specific procedures including STA-MCA bypass, hematoma evacuation, and ventricular drainage. Patient outcomes varied, with improvement (I), stability (S), deterioration (D), and death occurring in some cases (Table 16).

**Table 16:** Summary of management and outcomes of each reported patient. I: improvement, D: deterioration, S: static progression. Arficho et al. [6], Moramattom et al. [7], Mun et al. [8], Dou et al. [9], Dhawan et al. [10], Yamanaka et al. [11], Jindal et al. [12], Hammond et al. [13], Pinardi et al. [14], Galati et al. [15], Cadenas et al. [16],  Morino et al. [17], West et al. [18], Pugin et al. [19].

Study	Age (Years) and Gender	Type of Management (Medical or Surgical)	Drugs Administered	Type of Surgery	Patient Outcome (I/S/D, Death)
Arficho et al. [6]	55/M	Medical	Nil		S
Maramattom et al. [7]	36/F	Medical, surgery	Amphotericin B, fluconazole, antiplatelet, heparin	Left STA‐MCA bypass surgery with the end to side anastomosis. MCA stenting	Death
Mun et al. [8]	38/F	Surgery	Nil	Hematoma evacuation, cranioplasty with mesh plate, external ventricular drainage tube insertion	D
Dou et al. [9]	1.67/F	Medicine	Ceftriaxone, dexamethasone	–	S
Dhawan et al. [10]	7/F	Medicine	Vancomycin, ceftriaxone, meropenem, aspirin	–	D
Yamanaka et al. [11]	10/F	Medicine, surgery	Antiretroviral therapy	Bilateral ventricular drainage	Death
Jindal et al. [12]	10/M	Medicine	Aspirin	–	I
Hammond et al. [13]	11/F	Medicine	Aspirin	–	D
Hammond et al. [13]	10/M	Medicine	Antiretroviral therapy, aspirin	–	S
Hammond et al. [13]	5.8/F	Medicine	Antiretroviral therapy, aspirin	–	I
Hammond et al. [13]	3/F	Medicine	Antiretroviral therapy	–	D
Hammond et al. [13]	2.2/F	Medicine	Antiretroviral therapy, aspirin	–	S
Pinardi et al. [14]	55/M	Medicine	Aspirin, heparin, methylprednisolone, prednisone	–	I
Galati et al. [15]	65/M	Medicine	Antiretroviral therapy, antiplatelet, aspirin, warfarin	–	I
Cadenas et al. [16]	7/M	Surgery	Penicillin	Staged, bilateral, superficial temporal artery on–lay graft	I
Morino et al. [17]	2/M	Medicine	Acyclovir, aspirin	–	I
West et al. [18]	2.5/F	Medicine	Acyclovir, valaciclovir, aspirin	–	S
Pugin et al. [19]	53/M	Medicine	Ciprofloxacin, ceftriaxone, co-trimoxazole, vancomycin, rifampicin, methylprednisolone, prednisone	–	I

Patient outcomes varied in reported cases of post-infectious MMS. Improvement was most frequent (38.89%), followed by static condition (27.78%), deterioration (22.22%), and death (11.11%) (Table 17).

**Table 17:** Outcome of all reported patients, where the total number of patients N=18.

Patient Outcome	Number of Cases, n	Percentage Occurrence, n%
Improved	7	38.89%
Static	5	27.78%
Deteriorate	4	22.22%
Death	2	11.11%

Post-infectious MMS was associated with a range of severe neurological complications. Hemiplegia/hemiparesis was most frequent (44.44%), followed by ischemic strokes (27.78%), chronic infarcts (22.22%), and hemorrhage (22.22%). Additional complications, including facial palsy, hydrocephalus, seizures, and altered sensorium, also occurred (each at 11.11%) (Table 18).

**Table 18:** Complications encountered in reported patients, where the total number of patients N=18.

Complications	Number of Cases, n	Percentage Occurrence, n%
Hemiplegia/hemiparesis	8	44.44%
Ischemic strokes	5	27.78%
Chronic infarcts	4	22.22%
Hemorrhage	4	22.22%
Unspecified	3	16.67%
Facial palsy/weakness	2	11.11%
Hydrocephalus	2	11.11%
Convulsions/seizures	2	11.11%
Altered sensorium	2	11.11%
Recurrent transient ischemic attack	1	5.56%
Orthostatic transient ischemic spells	1	5.56%
Fever	1	5.56%
Coma	1	5.56%
Quadriparesis	1	5.56%
Speech impairment	1	5.56%
Ventricular rupture	1	5.56%
Encephalitis	1	5.56%
Motor aphasia	1	5.56%
Spasticity	1	5.56%
Frontal syndrome	1	5.56%
Dysphasia	1	5.56%
Dysmetria	1	5.56%
Relapsing painful scalp lesions	1	5.56%
Arterial stenosis/ vasculopathy	1	5.56%
Frontal lobe encephalomalacia	1	5.56%

Discussion

There are several pertinent theories that elucidate the hypothesized pathophysiology of post-infectious MMS. The Ring Finger Protein 213 (*RNF213*) gene has long been established as a genetic component for MMD due to its role in cerebrovascular angiogenesis and vascular remodeling [3]. However, recent studies have revealed *RNF213* to be involved in antimicrobial and immune regulatory processes as well [3]. Individuals with mutations in *RNF213* are already susceptible to idiopathic MMD; superimposed infections, such as human immunodeficiency virus (HIV) and bacterial meningitis, could serve as a second hit due to the profound inflammation and immune dysregulation they cause.

**Figure 1:**
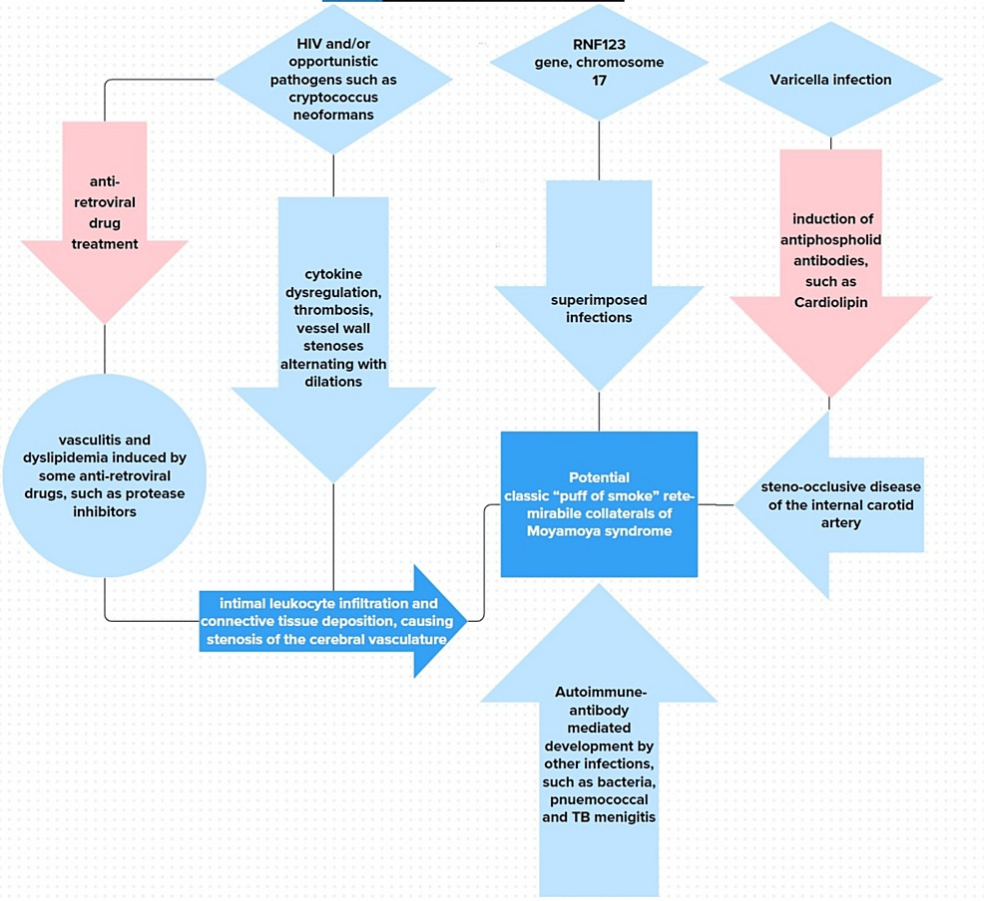
Pathophysiology of post-infectious Moyamoya syndrome proposed by the authors.

Moreover, another hypothesis explaining post-infectious MMS relates to post-infectious central nervous system vasculitis [6]. HIV and/or opportunistic pathogens such as *Cryptococcus neoformans* can infect endothelial cells of the cerebral vasculature, resulting in cytokine dysregulation, thrombosis, and vessel wall stenosis alternating with dilations [6]. The ensuing vascular inflammation leads to intimal leukocyte infiltration and connective tissue deposition, causing stenosis of the cerebral vasculature [7]. This lays the groundwork for the classic “puff of smoke” rete mirabile collaterals of MMS to develop (Figure 1).

It is of interest to note that a further cause of post-infectious MMS, specifically in HIV patients, could be a result of the vasculitis and dyslipidemia induced by some antiretroviral drugs, such as protease inhibitors [11]. One last theory pertains to the autoimmune effects of some infections. A Japanese study on the role of varicella infection and stroke secondary to MMS revealed the presence of antiphospholipid antibodies, especially anticardiolipin [17]. Well known to be thrombogenic, these antiphospholipid antibodies induced by varicella infection may have played a role in this patient’s steno-occlusive disease of the internal carotid artery [17]. Other infections could also elicit a similar autoimmune antibody-mediated development of post-infectious MMS.

Among the 14 articles we compiled for this narrative review, 55.56% of post-infectious MMS patients were female, and 44.44% were male. This is similar to the female sex predilection of MMD in the United States [1]. Moreover, a pedigree analysis of Japanese families has implied that epigenetic modification and imprinting might play a genetic role, as shown by a trend of maternal transmission to daughters [3]. Female predominance is also noted in other causes of MMS, such as neurofibromatosis, radiation, and hemoglobinopathies, with a female-to-male ratio as high as 3:1 in thalassemia patients [2].

The mean ages of adult and pediatric patients are 50.33 ± 10.19 years and 6.01 ± 3.48 years, respectively. This is consistent with an Italian cohort study on the pediatric population in which the average age of symptomatic MMS was 5.8 years [5]. There is likely not much significance to this age; in both our study and the aforementioned one, these ages simply lie near the middle of their respective age group ranges. Also, MMD and syndrome are known to have such a bimodal distribution.

The most common symptoms at presentation were hemiplegia/hemiparesis due to ischemic stroke (44.44% of patients) and fever (33.33%), followed by headache and altered sensorium (both at 27.78%). In the previously mentioned Italian study, 53.8% of patients with symptomatic MMS presented with ischemic stroke before diagnosis, and this was similarly the most common presentation [5]. Headache was reported in 18% of patients [5]. Among all those patients with MMS, 15.79% suffered from neurofibromatosis type I, 13.16% from sickle cell disease, and 21.05% from suspected genetic multisystemic syndrome [5]. Hemiplegia/hemiparesis most likely results from ischemic stroke since the hallmark of Moyamoya is cerebral vasculature steno-occlusion. Moreover, the thin-walled collaterals termed "rete mirabile" are prone to rupture, causing hemorrhage; headache is a common symptom of intracranial bleeding. However, in contrast to MMS due to non-infectious etiology, post-infectious MMS often presents with fever. This is logical considering the baseline infectious state and systemic inflammation present throughout the body.

The most frequently specified findings were hemiplegia and aphasia, both of which were identified in 11.11% of patients. An Indian case series on thalassemia and MMS revealed hemiplegia on examination in 75% of patients with MMS [2]. Likewise, a study on Pascual-Castroviejo type II syndrome (PHACE) syndrome examined a patient with aphasia and right hemiplegia who presented with MMS [20]. Finally, a Sudanese study on MMS involving a pediatric population with sickle cell disease revealed 91.3% of patients to have either right or left hemiplegia and 52.9% to exhibit aphasia [21]. Unlike hemiplegia, aphasia seems to be a rare manifestation of MMS secondary to non-infectious causes. This might be due to a relative sparing of the middle cerebral artery branches that supply Wernicke’s and Broca’s areas or that enough blood is supplied to these areas via collateral vessels to preserve speech function.

The most prevalent risk factor was HIV (50% of cases). Several other risk factors included chromosomal trisomy 21, Cryptococcal meningitis, and meningitis due to other organisms such as Mycobacterium tuberculosis and Pneumococcus (all at 11.11%). A portion of cases had no identified risk factors (11.11%). Since our study focused on infectious causes of MMS, we considered infections as risk factors. One of the most common disorders associated with MMS is Down syndrome [3]. In an American study on Down syndrome and MMS, 75% of patients had underlying structural heart disease such as ventricular septal defects and Tetralogy of Fallot, 44% had endocrine disorders such as hypothyroidism, and 38% had gastrointestinal disorders [4]. The study on PHACE syndrome stated that dysplasia and/or occlusion of major cerebral vessels, especially aortic coarctation, was a risk factor for developing MMS [22]. A case series studying radiation-induced MMS in children with brain tumors associated underlying neurofibromatosis as a risk factor for developing Moyamoya post-radiation syndrome, as well as younger age at irradiation [23]. The risk factors for this disease are diverse and multifaceted and vary greatly based on comorbidities.

In a significant portion of cases (44.44%), the time elapsed since presentation was unspecified. Among specified cases, presentation within days of symptom onset was the most common (33.33%). Presentations occurring weeks, months, or years after symptom onset were less frequent. The Italian study discussed earlier had 6.15% of patients reporting an isolated headache with an average of 32 months between headache onset and the diagnosis of MMS, while 10.77% reported a headache followed by other clinical symptoms with an average of 42 months between headache onset and the diagnosis of MMS [5]. This is similar to the study on thalassemia where neurological symptoms preceded diagnosis by an average of 35.57 ± 35.47 months (mean ± SD) [2]. Clearly, there is a discrepancy in the elapsed time between symptom onset and clinical presentation among MMS cases secondary to infection and secondary to other causes. Whereas the former presents within days, the latter presents much later. This could be due to the acute nature of infections such as pneumococcal meningitis, as shown in a case report on vasculitis secondary to pneumococcal meningitis where the symptom-presentation time was three days, including hospitalization [6]. The severe alteration of host immune responses and activation of proinflammatory pathways account for the rapid development of MMS in some infections [6].

Lesions were most frequent in the internal carotid artery and middle cerebral artery (both 66.66%) and anterior cerebral artery (50%). Lesions were less frequent in the posterior cerebral, vertebral, and basilar arteries. The frontal lobe (38.89%) and basal ganglia (33.33%) were commonly affected parenchymal regions. Other brain regions, such as the temporal lobe, ventricles, occipital lobe, and parietal lobe, were affected less frequently. Many cases demonstrated bilateral involvement (77.78%), with a smaller portion exhibiting right-sided lesions (22.22%). No cases were reported as solely left-sided lesions. These findings can be compared to the previously discussed Indian case series on thalassemia and MMS. One patient with beta-thalassemia major had an infarction in the right fronto-temporo-parieto-occipital region and right basal ganglia [2]. Two other patients demonstrated bilateral narrowing of the supraclinoid internal carotid artery (ICA), bilateral anterior cerebral artery (ACA), and middle cerebral artery (MCA) with corresponding involvement of the frontoparietal cortical and subcortical regions [2]. A final patient had bilateral terminal ICA narrowing with narrowing of the right M1 segment of MCA but also displayed left centrum semi-ovale infarction [2]. Although the majority of these findings are in concordance with our results, this study did demonstrate left-sided involvement, whereas ours did not. Furthermore, the study on Down syndrome and MMS showed that 59% of cases had bilateral involvement on presentation [4].

Fifty percent of the cases had normal hematological findings. A significant portion had unspecified hematological findings (33.33%). Less common findings included raised white blood cell count (particularly neutrophils), low CD4 count, and raised ASO titer/anti-DNase B (each at 5.56%). These findings are cause-specific: neutrophilia due to bacterial infections, low CD4 count due to HIV infection, etc. Similarly, a significant portion of blood cultures and serology lacked specific identification (38.89%). Normal findings were the next most common result (16.67%). Several infectious agents were detected, including Cryptococcal antigen (11.11%), unspecified Gram-positive cocci (11.11%), *Streptococcus pneumoniae* (5.56%), and VZV DNA + serum-VZV IgM (5.56%). Additionally, antibodies for Toxoplasma IgG and anti-double-stranded DNA IgG were present in 5.56% of cases each. Similarly, MMS due to other causes generally revealed unremarkable hematological findings. With the exception of hemolytic anemia in patients with sickle cell disease or thalassemia, most patients with MMS do not seem to have significant derangements in bloodwork. Moreover, culture and serological results were either not specified or were normal in the majority of MMS literature. One exception is the presence of the antiphospholipid antibodies, lupus anticoagulant, anticardiolipin, and anti-beta2 glycoprotein I, which were shown to be positive in some Down syndrome patients presenting with MMS [17]. This may be due to the various autoimmune conditions associated with Down syndrome, resulting in the production of thrombogenic antiphospholipid antibodies causing cerebral vascular occlusion and stenosis. This consistency between infectious and non-infectious causes of MMS seems to imply that significant systemic inflammation does not occur due to the vasculopathy, and if it does, it is a result of the underlying condition rather than Moyamoya.

Both CT and MRI showed evidence of restricted blood flow. CT findings indicated that stenosis, occlusion, and collateral formation were frequent vascular findings, although often unspecified in severity. Infarction, hypodensities, and hematoma were the most common parenchymal findings, with 22.22% of patients each showing evidence of such pathology. MRI findings revealed stenosis (50%) and collateral formation (44.44%) as the most prevalent vascular findings. Infarction was the most common parenchymal finding (66.66%). Hydrocephalus, encephalomalacia, and atrophy were less frequent. This is corroborated by a study that showed 74% of MMS (due to any cause) patients had bilateral steno-occlusions and collateral networks, 23% had unilateral steno-occlusion and collateral networks, and 3% had bilateral steno-occlusions and unilateral collateral networks [5]. This shows that neuroimaging does not differ significantly between the etiologies of MMS.

The vast majority of patients with post-infectious MMS received medical management alone (77.78%). A smaller proportion of patients underwent either surgical intervention alone (11.11%) or a combination of medical and surgical treatment (11.11%). Aspirin (50%) and antiretroviral therapy (38.89%) were the cornerstones of medical treatment for MMS. Antiretroviral therapy highlights the significance of HIV/AIDS as a risk factor. Ceftriaxone (16.67%) was another commonly administered antibiotic, and a substantial portion (16.67%) received unspecified medications. Various antiplatelet drugs, antibiotics, antifungals, and steroids were also used less frequently. Surgical management, which was less frequent, included specific procedures such as superficial temporal artery-middle cerebral artery (STA-MCA) bypass, hematoma evacuation, and ventricular drainage. In the Italian pediatric Moyamoya study, 69% of patients received aspirin and 73% underwent neurosurgery with either indirect or direct revascularization procedures [5]. In Down syndrome patients with MMS, 100% underwent surgery [4]. Among patients who developed MMS after radiation therapy, 26.6% underwent surgery while 20.2% received aspirin therapy [23]. These surgical rates are much higher than those in our study, where post-infectious MMS was preferentially treated with medical management alone. This could be due to the belief that treating or suppressing the underlying infection could result in clinical improvement without surgical intervention, such as controlling HIV viral load with antiretroviral therapy. Additionally, the fear of patient surgical complications while in an infectious state may have led clinicians to opt for the safer medical management route instead.

Patient outcomes varied in reported cases of post-infectious MMS. Improvement was most frequent (38.89%), followed by a static condition (27.78%), deterioration (22.22%), and death (11.11%). It was also associated with a range of severe neurological complications. Hemiplegia/hemiparesis was most frequent (44.44%), followed by ischemic strokes (27.78%), chronic infarcts (22.22%), and hemorrhage (22.22%). Additional complications, including facial palsy, hydrocephalus, seizures, and altered sensorium, also occurred (each at 11.11%). In contrast, 97% of Down syndrome patients treated for Moyamoya either improved or remained clinically stable, with 0% dying [4]. The most common complication was seizures at 9.8%, followed by stroke at 5.9% [4]. All other complications, including hemorrhage, were even lower. Among radiation-induced MMS patients, 24% improved and 24% remained stable post-surgery, with 12% dying and 4% deteriorating [23]. Stroke was the only major complication mentioned [23]. Seventy-six percent of pediatric patients with MMS improved after treatment (with a modified Rankin score, mRs, of <2), while 25% remained stable or deteriorated (mRs >2); no deaths occurred [5]. Six percent of patients suffered surgical complications in this study, including intraoperative hypoxic-ischemic damage, intraparenchymal bleed, and subdural hematoma, while medical management complications included adverse effects of aspirin such as petechial bleeding and cutaneous reactions observed in 6.67% of patients [5]. This variance in outcomes and complications between post-infectious and non-infectious MMS is likely due to the lower rates of surgery performed in the former, as well as clinical deterioration and death resulting from the underlying infection.

Limitations

We could not conduct a systematic review on this topic due to the scarcity of literature and preferred to include maximum published research studies. Findings of physical examination and culture and sensitivity, duration of presenting complaints, and follow-up details were missing in many research studies.

Clinical recommendations

**Figure 2:**
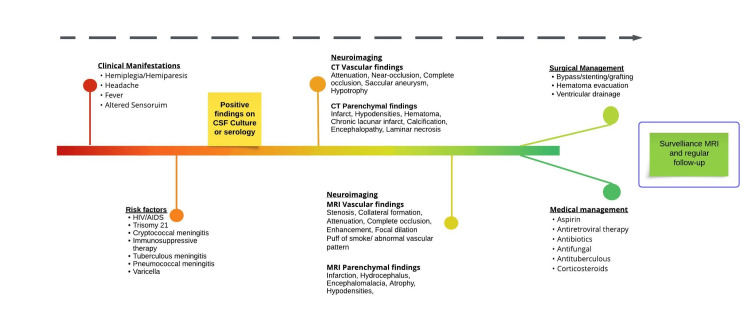
Schematic time-gradient disease course and management of post-infectious Moyamoya syndrome proposed by the authors.

All patients with MMS and MMD should be evaluated for possible ongoing infections, particularly for sequelae of infections such as HIV/AIDS, bacterial, tuberculous, and cryptococcal meningitis. Patients taking antiretroviral therapy should be screened for post-infective and post-treatment cerebral vascular changes, with surveillance using MRI (Figure 2). Early identification and treatment of infections can prevent long-term sequelae and possibly reverse vasculopathy. In the case of antiretroviral therapy, an early change of regimen can reduce or possibly prevent post-treatment vasculopathy. Genetic evaluation should be conducted, and family history should be investigated to identify patients who are more prone to developing the pathology.

## Conclusions

Post-infectious MMS is a unique and underappreciated variant of the broader MMD with its own demographics, clinical presentation, investigation findings, management, and outcomes. With a clear predilection towards affecting the pediatric population, especially those with HIV, post-infectious MMS might have been the real diagnosis in this population among those historically presenting with features of MMD. Since the treatment is preferentially medical management focused on addressing the causative agent, compared to surgical revascularization techniques for MMD and non-infectious MMS, complete knowledge and identification of this condition are imperative for clinicians. Investigational work-up for infectious pathology is warranted in cases of MMD. Further research is required to determine the extent to which management differs and how clinically significant the differences in etiology truly are.
